# Methylglyoxal‐induced apoptosis is dependent on the suppression of c‐FLIP_L_ expression *via* down‐regulation of p65 in endothelial cells

**DOI:** 10.1111/jcmm.13188

**Published:** 2017-04-26

**Authors:** Ji Hoon Jang, Eun‐Ae Kim, Hye‐Jin Park, Eon‐Gi Sung, In‐Hwan Song, Joo‐Young Kim, Chang‐Hoon Woo, Kyung‐Oh Doh, Kook Hyun Kim, Tae‐Jin Lee

**Affiliations:** ^1^ Department of Anatomy College of Medicine Yeungnam University Daegu South Korea; ^2^ Department of Pharmacology College of Medicine Yeungnam University Daegu South Korea; ^3^ Department of Physiology College of Medicine Yeungnam University Daegu South Korea; ^4^ Division of Gastroenterology and Hepatology Department of Internal Medicine Yeungnam University College of Medicine Daegu South Korea

**Keywords:** Methylglyoxal, HUVECs, c‐FLIP_L_, p65, ROS

## Abstract

Methylglyoxal (MGO) is a reactive dicarbonyl metabolite of glucose, and its plasma levels are elevated in patients with diabetes. Studies have shown that MGO combines with the amino and sulphhydryl groups of proteins to form stable advanced glycation end products (AGEs), which are associated with vascular endothelial cell (EC) injury and may contribute to the progression of atherosclerosis. In this study, MGO induced apoptosis in a dose‐dependent manner in HUVECs, which was attenuated by pre‐treatment with z‐VAD, a pan caspase inhibitor. Treatment with MGO increased ROS levels, followed by dose‐dependent down‐regulation of c‐FLIP_L_. In addition, pre‐treatment with the ROS scavenger NAC prevented the MGO‐induced down‐regulation of p65 and c‐FLIP_L_, and the forced expression of c‐FLIP_L_ attenuated MGO‐mediated apoptosis. Furthermore, MGO‐induced apoptotic cell death in endothelium isolated from mouse aortas. Finally, MGO was found to induce apoptosis by down‐regulating p65 expression at both the transcriptional and posttranslational levels, and thus, to inhibit c‐FLIP_L_ mRNA expression by suppressing NF‐κB transcriptional activity. Collectively, this study showed that MGO‐induced apoptosis is dependent on c‐FLIP_L_ down‐regulation *via* ROS‐mediated down‐regulation of p65 expression in endothelial cells.

## Introduction

Biological glycation has been investigated in the context of microvascular complications of diabetes, such as neuropathy, retinopathy and atherosclerosis 1. MGO, a reactive dicarbonyl compound, is formed during glycation and is also found in a range of beverages and foodstuffs, including freshly brewed coffee and carbonated soft drinks containing high‐fructose corn syrup 2, 3. MGO cross‐links with the amino groups of proteins to form irreversible AGEs, which leads to the cross‐linking or degradation of proteins 4. During glycation, MGO promotes the generation of reactive oxygen species (ROS) directly 5, 6. MGO is known to accumulate in various tissues at an accelerated rate under diabetic conditions, such as hyperglycaemia. Therefore, elevated MGO concentrations in patients with diabetes support the hypothesis that MGO contributes to diabetes‐associated vascular EC injury, which might be responsible for atherosclerosis 7, 8. The cytotoxic effects of MGO on tissues and cells have been attributed to its ability to induce apoptosis 9, 10. In addition, previous studies have shown that MGO induces apoptosis of human umbilical vein endothelial cells (HUVECs) by activating caspases, generating ROS, and activating MAP kinases 11, 12.

Although it is well established that MGO triggers ROS‐mediated apoptosis in HUVECs, no information is available regarding the regulatory proteins involved in ROS‐induced apoptosis. This study has been suggested that MGO might interfere with the expression of apoptotic regulatory proteins, which leads to MGO‐mediated apoptosis. To explore this, this study examined the effects of MGO on HUVECs by analysing the expression patterns and associated regulatory mechanisms of the regulators of apoptosis.

## Materials and methods

### Cells and materials

Human HUVECs were cultured in Clonetics^®^ EGM^®^ Endothelial Growth Medium (Lonza, Cologne, Germany) at 37°C in an atmosphere containing 5% (v/v) CO_2_. EA.hy926 cells (Rockville, MD, USA), a human EC line, were grown in Dulbecco's modified Eagle's medium (Gibco‐BRL, Gaithersburg, MD, USA) supplemented with 10% FBS and penicillin/streptomycin. The cells were maintained in a 37°C incubator with humidified 5% CO_2_. PCR primers were purchased from GenoTech (Daejeon, Korea). The anti‐c‐FLIP_L_ antibody was obtained from ALEXIS Corp. (San Diego, CA, USA). The anti‐PARP antibody was purchased from Cell Signalling Technology (Beverly, MA, USA). The anti‐actin antibody was acquired from Santa Cruz Biotechnology (Santa Cruz, CA, USA). MGO and other chemicals were supplied by Sigma‐Aldrich (St. Louis, MO, USA).

### Flow cytometry analysis

Cell counts were performed with a haemocytometer. Approximately 0.5 × 10^6^ cells were suspended in 100 μl of PBS, and 200 μl of 95% ethanol was added while vortexing. The cells were incubated at 4°C for 1 hr, washed with PBS and resuspended in 250 μl of 1.12% sodium citrate buffer (pH 8.4) together with 12.5 μg of RNase. Incubation was continued for 30 min. at 37°C. The cellular DNA was then stained by applying 250 μl of propidium iodide (50 μg/ml) for 30 min. at room temperature, and the relative DNA contents of the stained cells were analysed by fluorescence‐activated cell sorting (FACS) on a BD FACS Cato II flow cytometer (BD Biosciences, San Jose, CA, USA).

### Western blot analyses

The cellular lysates were prepared by suspending 1.2 × 10^6^ cells in 100 μl of lysis buffer (137 mM NaCl, 15 mM EGTA, 0.1 mM sodium orthovanadate, 15 mM MgCl_2_, 0.1% Triton X‐100, 25 mM MOPS, 100 μM phenylmethylsulphonyl fluoride and 20 μM leupeptin, adjusted to pH 7.2). The cells were disrupted by sonication and extracted at 4°C for 30 min. The proteins were electrotransferred to Immobilon‐P membranes (Millipore Corporation, Bedford, MA, USA), and the specific proteins were detected using an ECL Western blotting kit (Thermo Fisher Scientific, Rockford, IL, USA) according to the manufacturer's instructions.

### Transfection

The cells were plated onto 6‐well plates at a density of 0.5 × 10^6^ cells/well, grown overnight, and then cotransfected with 2 μg of various plasmid constructs and 1 μg of pCMV‐β‐galactosidase plasmid for 5 hrs using the Lipofectamine method. After transfection, the cells were cultured in 10% FBS medium with the vehicle (DMSO) or a drug for 24 hrs. HUVECs were transfected according to the recommendations in the optimized protocol (Amaxa, Gaithersburg, MD, USA).

### RNA isolation and reverse transcriptase PCR

The expression of c‐FLIP_L_ mRNA was determined by RT‐PCR. The total cellular RNA was extracted from cells using an EasyBlue reagent (Life Technologies, Seongnam, Korea), and the cDNA was prepared using an M‐MLV reverse transcriptase (Gibco‐BRL) according to the manufacturer's instructions. In addition, the total cellular RNA was reverse‐transcribed with a random primer and then amplified by PCR. GAPDH was used as the internal control. The following primers were used to amplify c‐FLIP_L_ and GAPDH: for c‐FLIP_L_, 5′‐CGGACTATAGAGTGCTGATGG‐3′ (sense) and 5′‐GATTAT CAGGCAGATTCCTAG‐3′ (antisense); for p65, 5′‐GACCTTTTCAACTTGGCTTCC‐3′ (sense) and 5′‐TGATGCTGTGGTCA GAAGGA‐3′ (antisense); and for GAPDH, 5′‐AGGTCGGAGTCAACGGATTTG‐3′ (sense) and 5′‐GTGATGGCATGG ACTGTGGT‐3′ (antisense). The PCR products were analysed by electrophoresis in 1.5% agarose gels and detected using UV light.

### 4′,6′‐Diamidino‐2‐phenylindole (DAPI) staining and TUNEL assay

The cells were fixed with 1% paraformaldehyde on a glass slide for 30 min. at room temperature. After washing with PBS, 300 nM 4′,6′‐diamidino‐2‐phenylindole (Roche, Mannheim, Germany) was added to the fixed cells for 5 min. The cells were then examined under a fluorescence microscope. TUNEL staining was performed with an *In Situ* Cell Death Detection Kit (Roche). All measurements were performed in a blinded manner, and at least three independent experiments were conducted.

### Cell death assessment by DNA fragmentation assays

A Cell Death Detection ELISA^PLUS^ kit (Roche Applied Science), which detects fragmented nuclear DNA, was used to assess the apoptotic activity. Briefly, culture plates were centrifuged for 10 min. at 200 g, the supernatants were removed, and pellets were lysed for 30 min. After centrifuging the plates at 200 g×g for 10 min., the collected supernatants containing the cytoplasmic histone‐associated DNA fragments were incubated with biotinylated histone antibody and peroxidase‐tagged mouse anti‐human DNA. After incubation with a peroxidase substrate for 5 min., the absorbance of the samples was measured at 405 and 490 nm (reference wavelength) using a microplate reader (A‐5082, Tecan, Mannedorf, Switzerland). The absorbance was corrected by subtracting the mean absorbance of the wells containing only the substrate. The results were expressed as the fold increase in the optical density of the treated sample to that of the untreated control.

### Measurement of ROS

The cells were incubated with MGO for 18 hrs, stained with 10 μM H_2_DCFDA for 40 min. at 37°C and observed by fluorescence microscopy (Axiovert 200M, Carl, Zeiss, Dublin, California, USA). The cells were incubated with MGO for 18 hrs and loaded with 10 μM H_2_DCFDA for 40 min. prior to harvesting. The fluorescence was measured at the desired time intervals by flow cytometry. The ROS generation was assessed by the dichlorofluorescein fluorescence intensity (FL‐1, 530 nm) of 10,000 cells with a FACScan flow cytometer (Becton‐Dickinson, San Jose, CA, USA).

### En face experiments and apoptosis assay

To determine the role of MGO in EC apoptosis *ex vivo*, C57BL/6 mice were anaesthetized, and the aortas were isolated. The isolated aortas were cultured in DMEM (Gibco) supplemented with 10% FBS, 50 units/ml penicillin and 50 μg/ml streptomycin. The aortas were treated with MGO for 18 hrs, fixed with 4% paraformaldehyde for 5 min. and permeabilized with PBS containing 0.1% Tween. The vascular fat was removed, and 5% goat serum was then used for blocking. The aortic ECs were then stained with antivascular endothelial‐cadherin antibody, which stains the EC junctions, overnight at 4°C. After washing with PBST, the cells were incubated with fluorescein isothiocyanate‐conjugated anti‐rat IgG (Invitrogen, Carlsbad, CA, USA) for 90 min. The cells were then stained with propidium iodide, and the apoptotic cells were observed by confocal microscopy (Leica, Bannockbum, IL, USA). All surgical procedures, including the isolation of aorta and en face immunofluorescence staining, were approved by the Institutional Animal Care and Use Committee (IACUC) of Yeungnam University College of Medicine.

### Statistical analysis

Statistical analysis was performed with a two‐tailed unpaired Student's *t*‐test for the two groups or using anova for three or more groups. At least three independent experiments were performed. The results are expressed as the means ± S.D.s, and *P* values of <0.05 were considered significant.

## Results

### MGO‐induced apoptosis in a dose‐dependent manner in HUVECs

**Figure 1:**
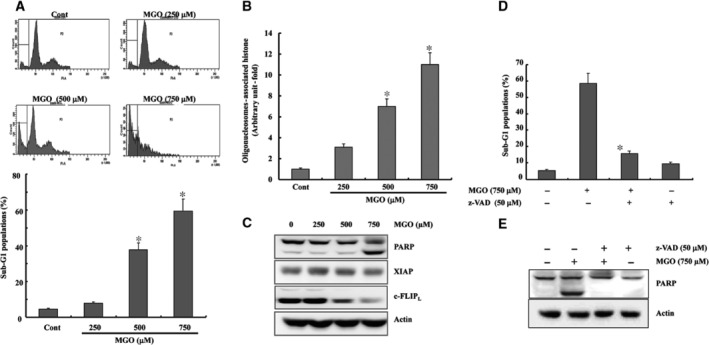
Methylglyoxal‐induced apoptosis in a dose‐dependent manner in HUVECs. (**A**) HUVECs were treated with the indicated concentration of MGO for 18 hrs, and the DNA contents of the treated cells were evaluated after propidium iodide staining. Apoptosis was measured as a sub‐G1 fraction by FACS. The FACS data are shown in the upper panel. The results are presented as the means ± S.D.s of three independent experiments, and bars represent standard deviations. *P < 0.05 *versus* non‐treated cells. (**B**) Cytoplasmic histone‐associated DNA fragmentation in the cells treated for 18 hrs with the indicated concentrations of MGO. A DNA fragmentation assay was performed as described in Materials and methods. The results are reported as the means of three independent experiments. *P < 0.05 *versus* non‐treated cells. (**C**) The cells were treated with the indicated concentrations of MGO. Equal amounts of cell lysates (40 μg) were subjected to electrophoresis and analysed for PARP, c‐FLIP_L_ and actin (for normalization) by Western blotting. (**D**) The HUVECs were incubated with 50 μM z‐VAD‐fmk or solvent for 1 hr before treatment with MGO for 18 hrs. Apoptosis was measured as a sub‐G1 fraction by FACS. The results are the means of three independent experiments, and the bars represent the standard deviations. *P < 0.05 *versus* MGO‐treated cells. (**E**) Equal amounts of cell lysates (40 μg) were subjected to electrophoresis and analysed by Western blotting for PARP. Actin was used the loading control.

To determine the cytotoxic effects of MGO on HUVECs, the cells were treated with various concentrations of MGO (250–750 μM) to reflect pathological conditions, because the concentration of MGO in the blood has been reported to be ~400 μM in patients with diabetes 13, 14. As shown in Figure 1A, treatment of ECs with MGO resulted in a marked and dose‐dependent increase in sub‐G1 phase accumulation. The proapoptotic effect of MGO on HUVECs was further confirmed by a TUNEL assay (Fig. 1B). The involvement of caspases in MGO‐induced cell death was examined, and treatment with MGO activated caspase‐related events, such as the cleavage of PARP (Fig. 1C). In addition, MGO‐induced cell death was prevented by pre‐treating the cells with z‐VAD‐fmk (a non‐specific caspase inhibitor), as determined by FACS and PARP cleavage (Fig. 1D and E). These results suggest that MGO‐induced cell death was mediated by caspase‐dependent cell death pathways in HUVECs. The underlying mechanism involved was examined by analysing the expression levels of various apoptosis‐regulating proteins using a Western blot assay. As shown in Figure 1C, the level of c‐FLIP_L_ protein was dose‐dependently reduced in response to MGO treatment in HUVECs.

Because c‐FLIP is a key regulator that determines the activity of caspase‐8 15, the procaspase‐8 levels were checked after MGO treatment in HUVECs. As shown in Figure S1A, treatment with MGO also caused processing of procaspases‐8, resulting in the appearance of p42/41‐kD fragments. The suppression of capase‐8 expression by siRNA partly inhibited MGO‐induced apoptosis in HUVECs (Fig. S1B). These observations suggest that capase‐8 activation was partly involved in MGO‐mediated apoptosis in HUVECs.

### MGO‐induced apoptosis was dependent on ROS generation caused by the down‐regulation of c‐FLIP_L_ expression

**Figure 2:**
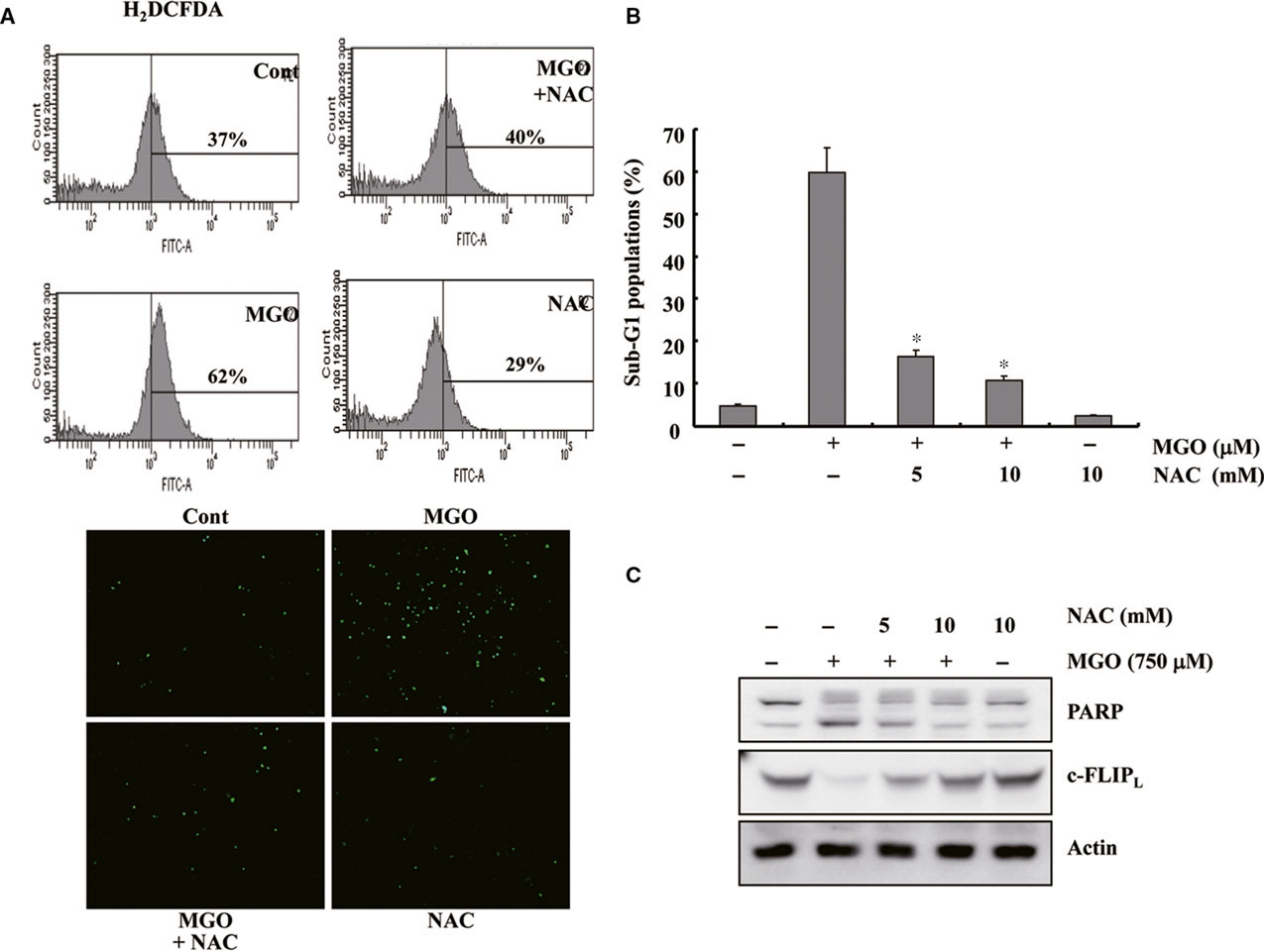
MGO‐induced apoptosis was dependent on the generation of ROS and subsequent down‐regulation of c‐FLIP_L_ protein. (**A**) HUVECs were treated in the presence or absence of NAC (10 mM). The cells were loaded with H_2_DCFDA, a fluorescent dye and stimulated with MGO in the presence or absence of NAC (5 and 10 mM). H_2_DCFDA fluorescence was visualized by flow cytometry (top) or fluorescence microscopy (bottom). (**B**) Apoptosis was analysed as a sub‐G1 fraction by FACS. *P < 0.05 *versus* MGO‐treated cells. (**C**) Pre‐treatment with NAC attenuated the MGO‐induced cleavage of PARP and the down‐regulation of c‐FLIP_L_ protein. The cells were treated with MGO in the presence of the indicated concentrations of NAC (5 or 10 mM). Western blotting analysis was performed as described in the legend of Fig. 1.

Previous studies have shown that MGO can induce apoptosis by promoting ROS production in different cell types 16, 17. Therefore, this study examined whether MGO induces ROS production in HUVECs using H_2_DCFDA‐derived fluorescence. As shown in Figure 2A, MGO markedly increased the intracellular ROS levels. To determine whether ROS generation plays a role in MGO‐induced apoptosis, HUVECs were pre‐treated with N‐acetyl‐l‐cysteine (NAC, an ROS scavenger). NAC attenuated the MGO‐mediated apoptosis, PARP cleavage and ROS generation (Fig. 2A and B). Furthermore, ROS scavengers prevented the down‐regulation of c‐FLIP_L_ in MGO‐treated cells (Fig. 2C). These observations suggest that ROS play a critical role in MGO‐mediated apoptosis by down‐regulating c‐FLIP_L_ expression.

### Overexpression of c‐FLIP_L_ partially attenuated MGO‐induced apoptosis

**Figure 3:**
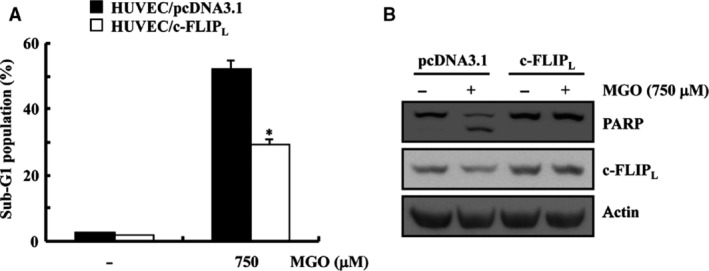
Overexpression of c‐FLIP_L_ partially attenuated MGO‐induced apoptosis. (**A**) HUVECs/pcDNA3.1 and HUVECs/c‐FLIP_L_ were treated for 18 hrs with MGO. Apoptosis was assessed by determining the proportion of cells in the sub‐G1 fraction by FACS. The results are presented as the means ± S.D.s (*n* = 3). *P < 0.05 *versus* MGO‐treated pcDNA3.1 cells. (**B**) Equal amounts of cell lysates (40 μg) were electrophoresed and analysed by Western blotting.

This study examined whether the down‐regulation of c‐FLIP_L_ by MGO is essential to stimulate apoptosis in HUVECs. Overexpression of c‐FLIP_L_ significantly attenuated MGO‐facilitated apoptosis, while treatment with MGO‐induced significant apoptosis in the empty vector‐transfected HUVECs (Fig. 3A). PARP cleavage was also analysed in both cell lines. As shown in Figure 3B, ectopic expression of c‐FLIP_L_ reduced PARP cleavage induced by MGO treatment in HUVECs. These results indicate that down‐regulation of c‐FLIP_L_ contributes to MGO‐induced apoptosis in HUVECs.

### MGO‐induced c‐FLIP_L_ down‐regulation was mediated by inactivation of the NF‐κB pathway

**Figure 4:**
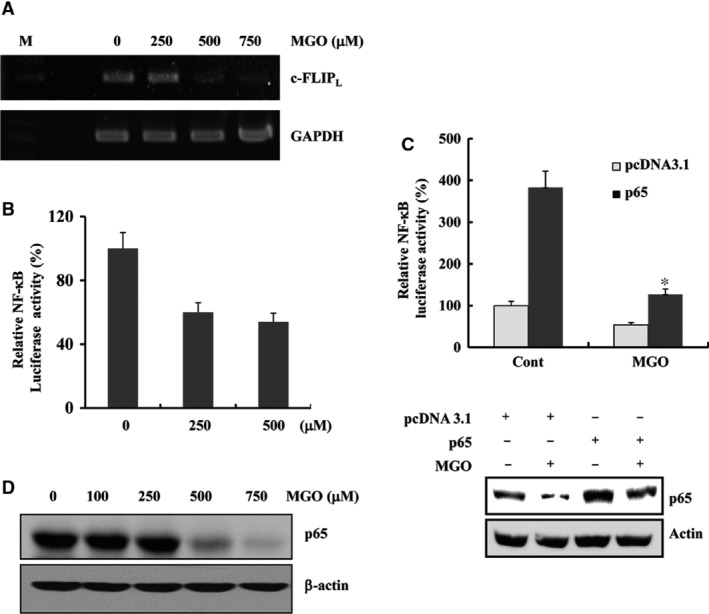
MGO‐induced c‐FLIP_L_ down‐regulation was mediated by inactivation of the NF‐κB pathway**.** (**A**) HUVECs were treated with the indicated concentrations of MGO for 18 hrs. The total RNA was isolated, and RT‐PCR analysis was performed, as described in Materials and methods. A representative study is shown; two additional experiments yielded similar results. (**B**) The cells were transfected with an NF‐κB reporter plasmid and then treated with MGO at the indicated concentrations for 18 hrs. The cell lysates were assayed for luciferase activity using a luminometre. The transfection efficiency differences were normalized by cotransfecting with a LacZ‐containing plasmid. (**C**) After transient transfection with empty vector or the p65 expression vector, the cells were treated with MGO for 18 hrs. The luciferase activities were measured as described above. *P < 0.05 forMGO‐treated pcDNA3.1 transfectants *versus* MGO‐treated p65 transfectants. Equal amounts of cell lysates (40 μg) were electrophoresed and Western blotted (bottom). (**D**) HUVECs were treated with the indicated concentrations of MGO for 18 hrs. Equal amounts of cell lysates (40 μg) were electrophoresed and analysed by Western blotting for p65 and actin (for normalization purposes).

The c‐FLIP_L_ mRNA levels were examined by RT‐PCR to further elucidate the mechanism responsible for the changes in expression of the c‐FLIP_L_ protein. As shown in Figure 4A, MGO treatment of HUVECs reduced the mRNA levels of c‐FLIP_L_ in a dose‐dependent manner, suggesting that the MGO‐mediated down‐regulation of c‐FLIP_L_ protein was regulated at the transcriptional level.

Because c‐FLIP_L_ can be transcriptionally regulated through the nuclear factor‐κB pathway 18, an NF‐κB gene‐dependent reporter assay was performed with a pNF‐κB‐Luc plasmid containing four NF‐κB binding sites. The HUVECs were transiently transfected with the pNF‐κB‐Luc plasmid and then stimulated with MGO. As shown in Figure 4B, the MGO treatment significantly reduced NF‐κB‐dependent luciferase activity. The functional significance of NF‐κB in the down‐regulation of c‐FLIP_L_ in MGO‐treated cells was next examined. Ectopic expression of p65 by transient transfection markedly blocked the down‐regulation of c‐FLIP_L_ induced by MGO treatment (Fig. 4C). Interestingly, the MGO treatment also reduced the p65 protein levels in HUVECs (Fig. 4D). These results suggest that the MGO‐induced c‐FLIP_L_ down‐regulation in HUVECs might be caused by inhibition of the NF‐κB pathway.

### Low‐dose MGO‐induced apoptosis in HUVECs and in endothelium isolated from mouse aorta

**Figure 5:**
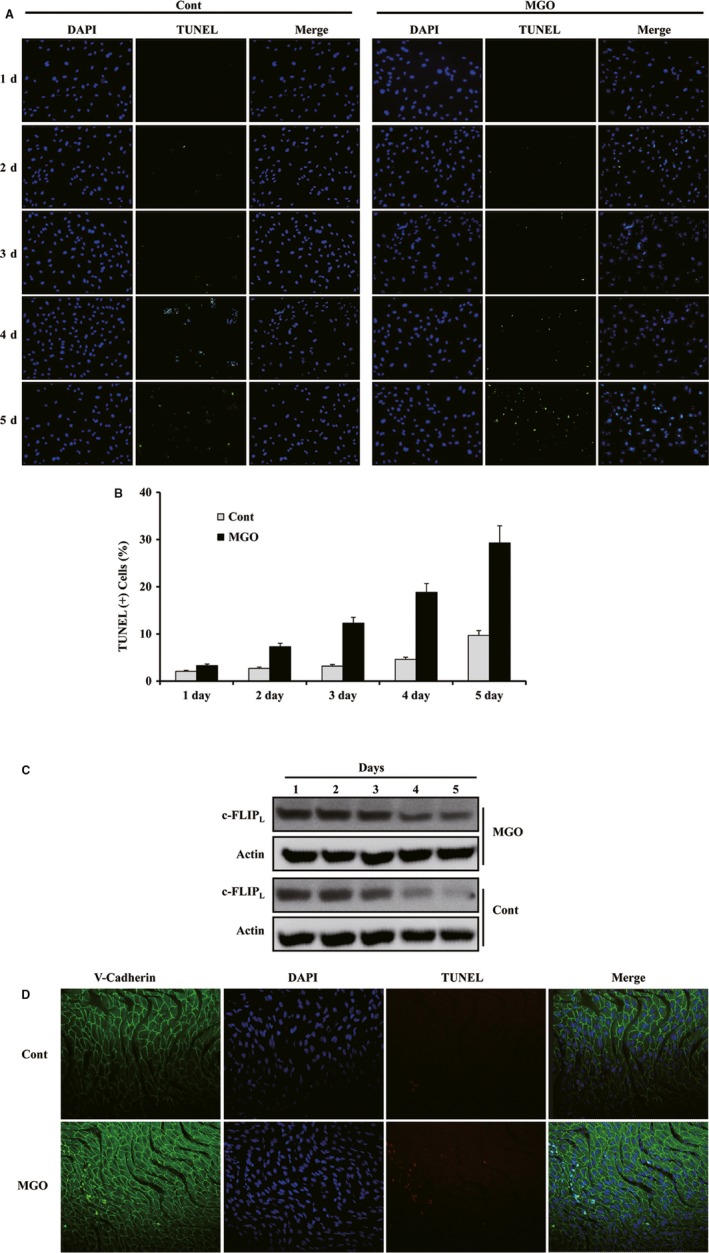
Low‐dose MGO‐induced apoptosis in HUVECs and in endothelial cells (ECs) isolated from mouse aortas. (**A**) HUVECs were treated with MGO (100 μΜ) for the indicated times. The cell nuclei were stained with TUNEL and DAPI and then observed by fluorescence microscopy at a magnification of ×200. TUNEL‐positive cells are shown as green. No treatment is given to the control. (**B**) The percentages of TUNEL‐positive cells. The experiments were repeated three times. (**C**) Equal amounts of cell lysates (40 μg) were electrophoresed and Western blotted for c‐FLIP_L_ and actin (for normalization). (**D**) The isolated aortas were treated with 100 μM MGO for 18 hrs, and after fixing, the aortic ECs were stained with antivascular endothelial‐cadherin antibody and then DAPI and TUNEL stained.

To determine whether prolonged exposure to a low dose of MGO could induce apoptosis in HUVECs, cells were exposed to 100 μM of MGO for 1 to 5 days. As shown in Figure 5A and B, TUNEL‐positive cells were markedly detected after 3 days of MGO treatment. Western blotting analysis showed that the MGO treatment resulted in time‐dependent reductions in the c‐FLIP_L_ protein levels (Fig. 5C). To determine whether MGO induces apoptosis in ECs *ex vivo*, aortas isolated from mice were treated with 100 μM MsGO for 18 hrs. The en face preparations of aortas were stained with propidium iodide and antivascular endothelial‐cadherin antibody. Staining showed that exposure to low‐dose MGO enhanced apoptosis in ECs for 18 hrs (Fig. 5D). These results indicate that prolonged exposure to low doses of MGO caused apoptosis in HUVECs and the endothelium and suggest that pathological concentrations of MGO in the blood causes endothelium damage in patients with diabetes.

### MGO‐induced apoptosis was dependent on down‐regulation of c‐FLIP_L_ by ROS generation in EA.hy926 cells

**Figure 6:**
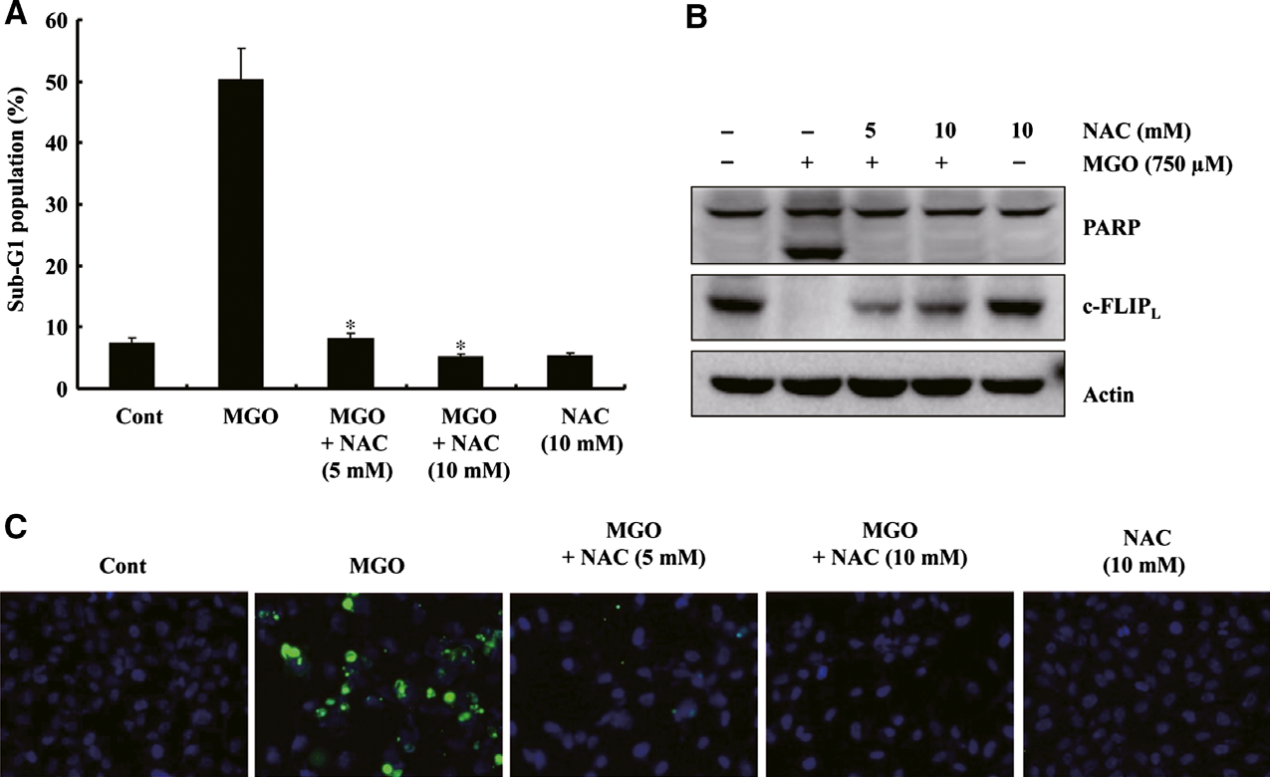
MGO‐induced apoptosis was dependent on the generation of ROS and the subsequent down‐regulation of c‐FLIP expression in EA.hy926 cells. (**A**) Apoptosis was assessed by determining the proportion of cells in the sub‐G1 fraction by FACS. *P < 0.05 for MGO+NAC‐treated cells *versus* MGO‐treated cells. (**C**) The cells were treated with MGO at the indicated concentrations and NAC (5 or 10 mM). Western blotting was performed as described above. The EA.hy926 cells were treated with MGO in the presence or absence of NAC, TUNEL stained, and then, observed by fluorescence microscopy at a magnification of ×200. The TUNEL‐positive cells are shown as green. (B) Pretreatment with NAC attenuated the MGO‐induced cleavage of PARP and the down‐regulation of c‐FLIPL protein. The cells were treated with MGO in the presence of the indicated concentrations of NAC (5 or 10 mM). Western blotting analysis was performed as described in the legend of Fig. 1.

To examine the mechanism responsible for the MGO‐induced down‐regulation of c‐FLIP_L_, another *in vitro* model of ECs was used, that is transformed human umbilical vein ECs (EA.hy926). As shown in Figure 6A and B, NAC‐attenuated MGO‐mediated apoptosis and PARP cleavage. Furthermore, ROS scavengers prevented the down‐regulation of c‐FLIP_L_ in the MGO‐treated EA.hy926 cells (Fig. 6B). As shown in Figure 6C, the MGO treatment enhanced the number of TUNEL‐positive cells, which were reduced by NAC pre‐treatment. These results support the notion that ROS play a critical role in MGO‐mediated apoptosis by down‐regulating c‐FLIP_L_ expression.

### MGO‐induced c‐FLIP_L_ down‐regulation was mediated by the suppression of p65 expression

**Figure 7:**
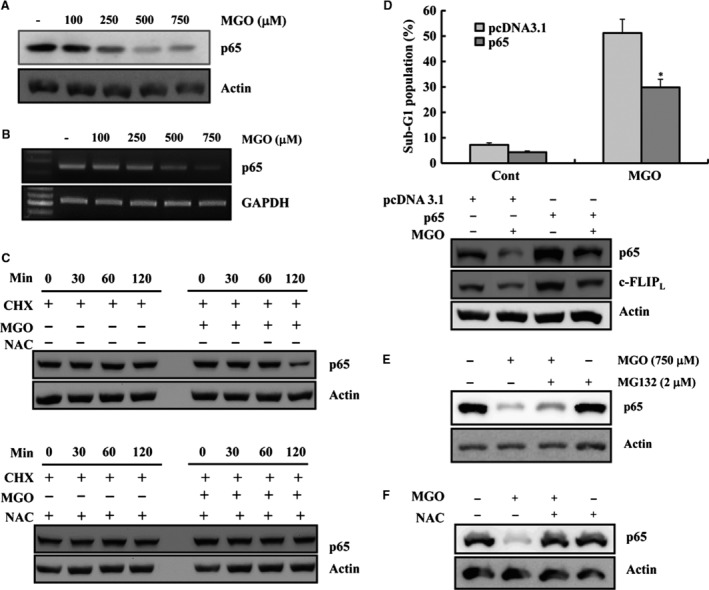
MGO‐induced c‐FLIP_L_ down‐regulation was mediated by the suppression of p65 expression. (**A**) EA.hy26 cells were treated with the indicated concentrations of MGO for 18 hrs. Equal amounts of cell lysates (40 μg) were electrophoresed and Western blotted as described above. (**B**) The cells were treated with the indicated concentrations of MGO for 18 hrs. The total RNA was isolated, and RT‐PCR was performed, as described in Materials and methods. A representative study is shown; two additional experiments yielded similar results. (**C**) EA.hy926 cells were treated with MGO in the presence or absence of CHX for the indicated times (upper panel). NAC plus CHX in the absence or presence of MGO (upper panel). Western blotting was performed with anti‐p65 and anti‐actin antibody (actin served as the loading control). (**D**) After transient transfection with the empty vector or p65 expression vector, the cells were treated with MGO for 18 hrs. Apoptosis was assessed by determining the proportion of cells in the sub‐G1 fraction by FACS. *P < 0.05 *versus* MGO‐treated pcDNA3.1 cells (upper). Equal amounts of cell lysates (40 μg) were electrophoresed and Western blotted (bottom). (**E**) EA.hy26 cells were treated with 750 μM MGO in the presence or absence of 2 μM MG132 for 18 hrs. Western blotting was performed with anti‐p65 or anti‐actin antibodies (actin served as the protein loading control). (**F**) EA.hy26 cells were treated with MGO in the presence or absence of NAC for 18 hrs. Western blotting was performed with the anti‐p65 antibody or anti‐actin antibody (actin served as the loading control).

Previous studies reported that down‐regulating the expression of the p65 subunit of NK‐κB inhibits proliferation by inducing apoptosis in cancer cells 19, 20, 21. In addition, MGO reduced the p65 protein levels in EA.hy926 cells (Fig. 7A). To further elucidate the mechanism of p65 reduction, p65 mRNA levels were examined by RT‐PCR, and a protein stability test for p65 was performed. As shown in Figure 7B, MGO dose‐dependently decreased the c‐FLIP_L_ mRNA levels in EA.hy926 cells, suggesting that the MGO‐mediated down‐regulation of p65 protein might be regulated at the transcriptional level. The EA.hy926 cells were then treated with cycloheximide (CHX) and MGO, as indicated in Figure 7C, and the degradation of the p65 protein was facilitated by MGO treatment (Fig. 7C, upper panel), indicating that MGO reduces the stability of the p65 protein. Interestingly, we found that ROS scavengers prevented the degradation of the p65 protein facilitated by MGO treatment, supporting that ROS seem to play role of MGO‐mediated p65 protein degradation (Fig. 7C, lower panel). Furthermore, ectopic expression of p65 by transient transfection markedly blocked MGO‐induced apoptotic cell death and prevented c‐FLIP_L_ protein down‐regulation by MGO (Fig. 7D).

To determine whether p65 protein is degraded *via* the proteasome‐dependent pathway, cells were treated with MGO in the absence or presence of a proteasome inhibitor, MG132 (2 μM). As expected, the decreased protein level of p65 in the MGO‐treated EA.hy926 cells was partly recovered to basal levels by MG132 treatment (Fig. 7E). These results indicate that the MGO‐mediated down‐regulation of c‐FLIP_L_ was caused by both a reduction in the stability of p65 protein and reduced p65 mRNA transcription in ECs. Because ROS scavengers reversed the down‐regulation of c‐FLIP_L_ expression in MGO‐treated cells (Fig. 6B), this study tested whether the down‐regulation of p65 expression was prevented by NAC pre‐treatment. As shown in Figure 7F, pre‐treatment with NAC restored p65 expression in MGO‐treated cells. Finally, to determine whether MGO‐mediated p65 down‐regulation was involved in c‐FLIP_L_ mRNA transcription, the c‐FLIP_L_ mRNA levels were examined by RT‐PCR and c‐FLIP_L_ promoter activity. Forced expression of p65 prevented MGO‐induced c‐FLIP_L_ mRNA down‐regulation and recovered c‐FLIP promoter activity that was reduced by MGO (Fig. S2). These results suggest that MGO‐induced apoptosis is caused sequentially by ROS generation, p65 down‐regulation, and the suppression of c‐FLIP_L_ expression in ECs. As inactivation of the Akt pathway mediates inhibition of p65 expression 22, we checked whether ectopic expression of dominant active Akt could block the MGO‐mediated p65 down‐regulation in HUVECs. As shown in Figure S3, overexpression of dominant active Akt blocked MGO‐induced apoptotic cell death and MGO‐inhibited p65 protein expression.

## Discussion

In the present study, MGO was found to be cytotoxic to both HUVECs and transformed human umbilical vein ECs, and MGO‐mediated apoptosis was found to be dependent on the generation of ROS and subsequent down‐regulation of the NF‐κB pathway and of c‐FLIP_L_ expression in human ECs.

MGO is a cytotoxic metabolite that is produced by impaired glucose metabolism. Under a number of pathological conditions, including diabetes and neurodegenerative disorders, the MGO levels are elevated in body fluids and tissues 23, 24. Furthermore, MGO exposure has been reported to provoke the generation of intracellular ROS and subsequent apoptosis in HUVECs 25, 26. In addition, MGO has been reported to suppress the expression of antiapoptotic proteins, such as Bcl‐2, Bcl‐X_L_ and XIAP (X‐linked inhibitor of apoptosis protein) 27, 28, and induce apoptosis. Therefore, this study investigated whether ROS generation occurs in MGO‐treated HUVECs and examined the cytotoxic effects of ROS. Consistent with previous observations, intracellular ROS were generated after treating HUVECs with MGO.

Studies on several ROS‐inducing agents have shown that ROS‐dependent suppression of antiapoptotic proteins, such as, c‐FLIP_L_, Mcl‐1, and Akt, result in cell death 29, 30. In line with these observations, MGO treatment resulted in the down‐regulation of c‐FLIP_L_ protein in ECs. In addition, in the presence of NAC, the reduction in the c‐FLIP_L_ protein level caused by MGO was prevented, indicating that ROS down‐regulated c‐FLIP expression in MGO‐treated ECs.

As c‐FLIP_L_ can be transcriptionally regulated by the NF‐κB pathway 31, 32, the p65 expression patterns after MGO exposure were checked using an NF‐κB gene‐dependent reporter assay. Interestingly, MGO down‐regulated c‐FLIP_L_ expression by suppressing p65 transcription and decreasing p65 protein stability in EA.hy926 cells. Treatment with low concentrations of MGO (<100 μM) increase p65 expression in ECs 11, 33, but at higher concentrations (>400 μM), MGO suppresses the NF‐κB signalling pathway by inhibiting NF‐κB DNA‐binding or by increasing the total IκBα levels 27, 28, 34. In the present study, a higher concentration of MGO‐induced apoptosis with concomitant inactivation of the NF‐κB signalling pathway by suppressing p65. Therefore, ECs can be treated with MGO concentrations >400 μM because the intracellular MGO concentrations are >300 μM, which is considerably higher than the plasma MGO concentrations because MGO is formed during glycolysis 35 and because only 1.8% of exogenous MGO was found to be taken up by the ECs 36. Accordingly, ECs were treated with MGO concentrations of 250 to 750 μM. Furthermore, in agreement with these findings, high levels of MGO have been reported to be associated with loss of cell viability and increased cell death 37, 38.

The processes associated with’ MGO elevation are believed to occur in patients with persistent hyperglycaemia and in the aortas of spontaneously hypertensive rats (SHR) 14, 39, 40. To examine the chronic effects of low concentrations (100 μM) of MGO, particularly in blood vessels, this study tested whether long‐term exposure to MGO at 100 μM could induce apoptosis in HUVECs. TUNEL‐positive cells were observed after 3 days of MGO exposure, and MGO caused time‐dependent reductions in the c‐FLIP_L_ protein levels and enhanced apoptosis in the mouse endothelium, suggesting that relatively low concentrations of MGO could induce endothelium damage in patients with diabetes.

**Figure 8:**
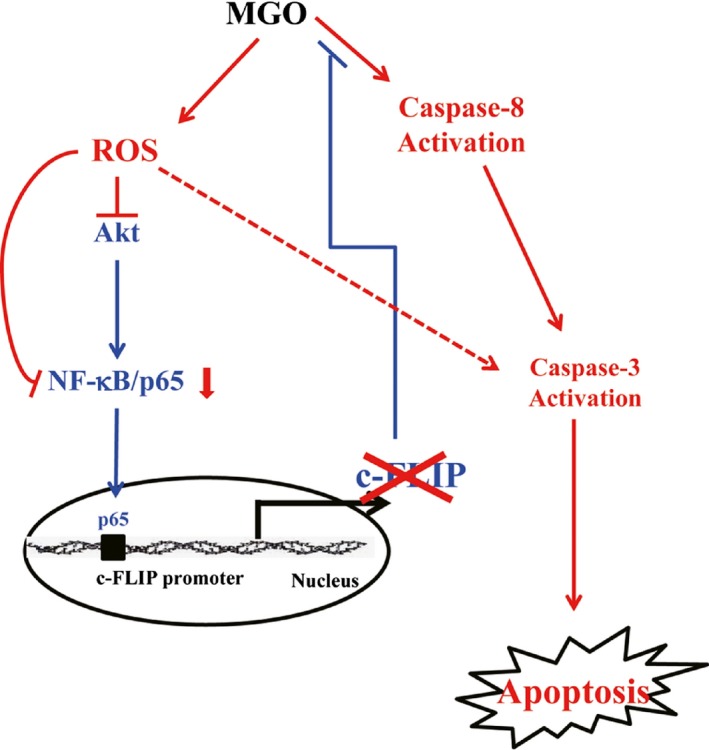
Schematic summary of MGO‐mediated cell death in endothelial cells.

In conclusion, MGO‐induced the generation of intracellular ROS, which repressed NF‐κB pathways by down‐regulating p65 expression, and in turn, down‐regulated antiapoptotic c‐FLIP_L_, leading to apoptosis in ECs (Fig. 8). These findings are expected to help reveal further mechanisms of physiological importance, especially regarding interactions between MGO and apoptosis‐regulating proteins.

## Conflict of interest

The authors have no conflict of interests to declare.

## Supporting information


**Fig. S1** (A) HUVECs were treated with the indicated concentrations of MGO. Equal amounts of cell lysates (40 μg) were subjected to electrophoresis and analyzed for caspase‐8 and actin (for normalization) by western blotting. p55 indicates procapsapase‐8. p43/41 indicate the cleaved capsapase‐8 fragments. (B) HUVECs were transfected with si‐c‐FLIP_L_ with si‐caspase‐8 or si‐Cont. Twenty‐four hours after transfection, the cells were treated with MGO for 18 hrs. Apoptosis was analyzed as the sub‐G1 fraction by FACS (left). *P < 0.05 compared to each MGO‐treated si‐Cont‐transfected cells. Immunoblots for caspase‐8 and actin antibodies (right). p55 indicates procapsapase‐8. p43/41 indicate the cleaved capsapase‐8 fragments.

**Fig. S2** (A) RT‐PCR analysis of c‐FLIP_L_ mRNA in EA.hy26 cells transfected as indicated. (B) EA.hy26 cells were transfected with a c‐FLIP promoter containing luciferase vector and then treated with MGO for 18 hrs. The cell lysates were assayed for the luciferase activity using a luminometer. The differences in transfection efficiency were normalized by cotransfecting with a LacZ‐containing plasmid. *P < 0.05 *versus* MGO‐treated pcDNA3.1 cells.

**Fig. S3** (A) EA.hy26/pcDNA3.1 and EA.hy26/DA‐Akt were treated for 18 hrs with MGO. Apoptosis was assessed by determining the proportion of cells in the sub‐G1 fraction by FACS. *P < 0.05 *versus* MGO‐treated pcDNA3.1 cells. (B) Equal amounts of cell lysates (40 μg) were electrophoresed and analyzed by Western blotting.
