# Supplementary figures and images for: Methylglyoxal‐induced apoptosis is dependent on the suppression of c‐FLIPL expression via down‐regulation of p65 in endothelial cells
Source: J Cell Mol Med. 2017 Apr 26;21(11):2720–31. doi: 10.1111/jcmm.13188 (PMC5661116; doi:10.1111/jcmm.13188)

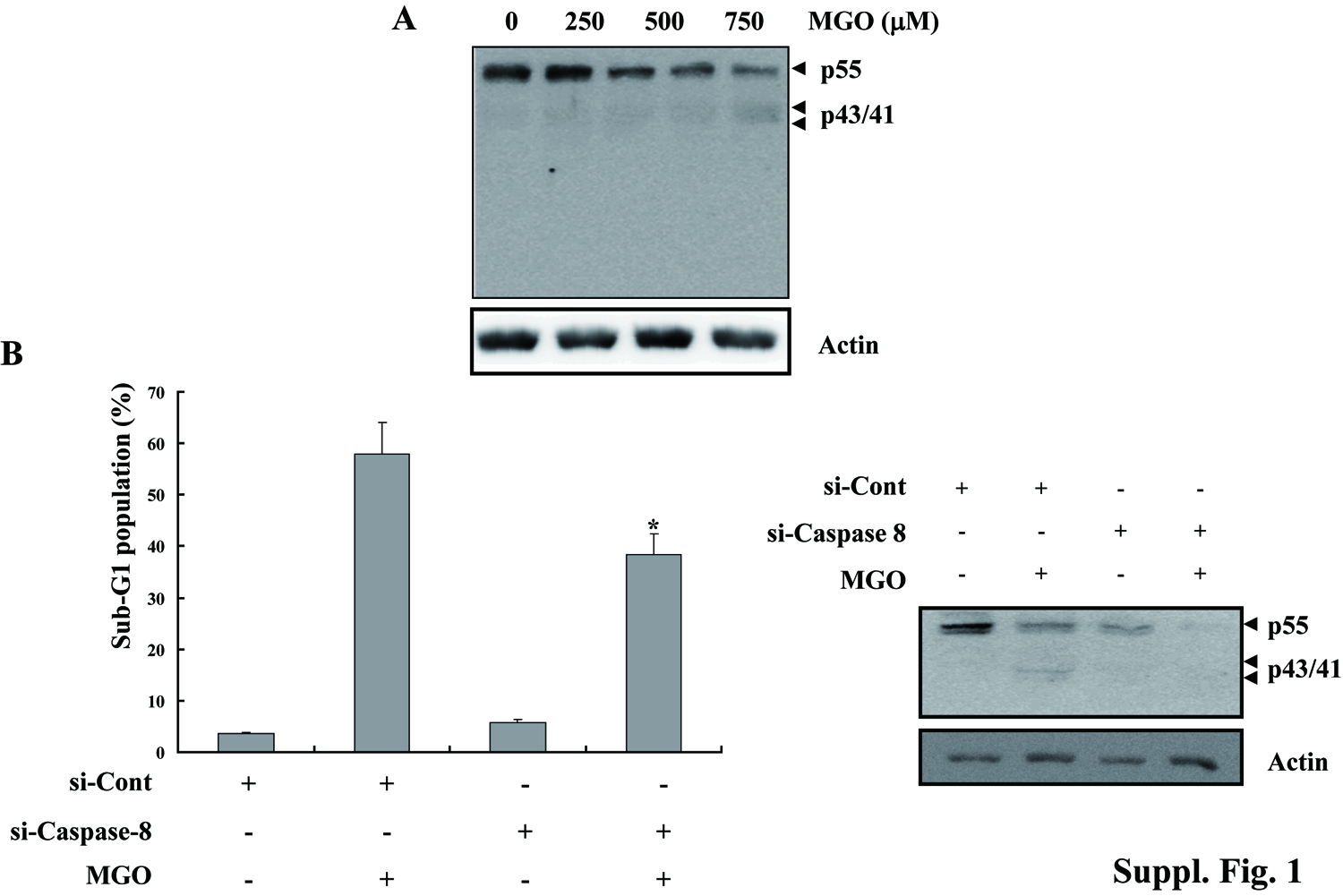

Supplement: Supplementary file 1 — Fig. S1 (A) HUVECs were treated with the indicated concentrations of MGO. Equal amounts of cell lysates (40 μg) were subjected to electrophoresis and analyzed for caspase‐8 and actin (for normalization) by western blotting. p55 indicates procapsapase‐8. p43/41 indicate the cleaved capsapase‐8 fragments. (B) HUVECs were transfected with si‐c‐FLIPL with si‐caspase‐8 or si‐Cont. Twenty‐four hours after transfection, the cells were treated with MGO for 18 hrs. Apoptosis was analyzed as the sub‐G1 fraction by FACS (left). *P < 0.05 compared to each MGO‐treated si‐Cont‐transfected cells. Immunoblots for caspase‐8 and actin antibodies (right). p55 indicates procapsapase‐8. p43/41 indicate the cleaved capsapase‐8 fragments. [file JCMM-21-2720-s001.tif]

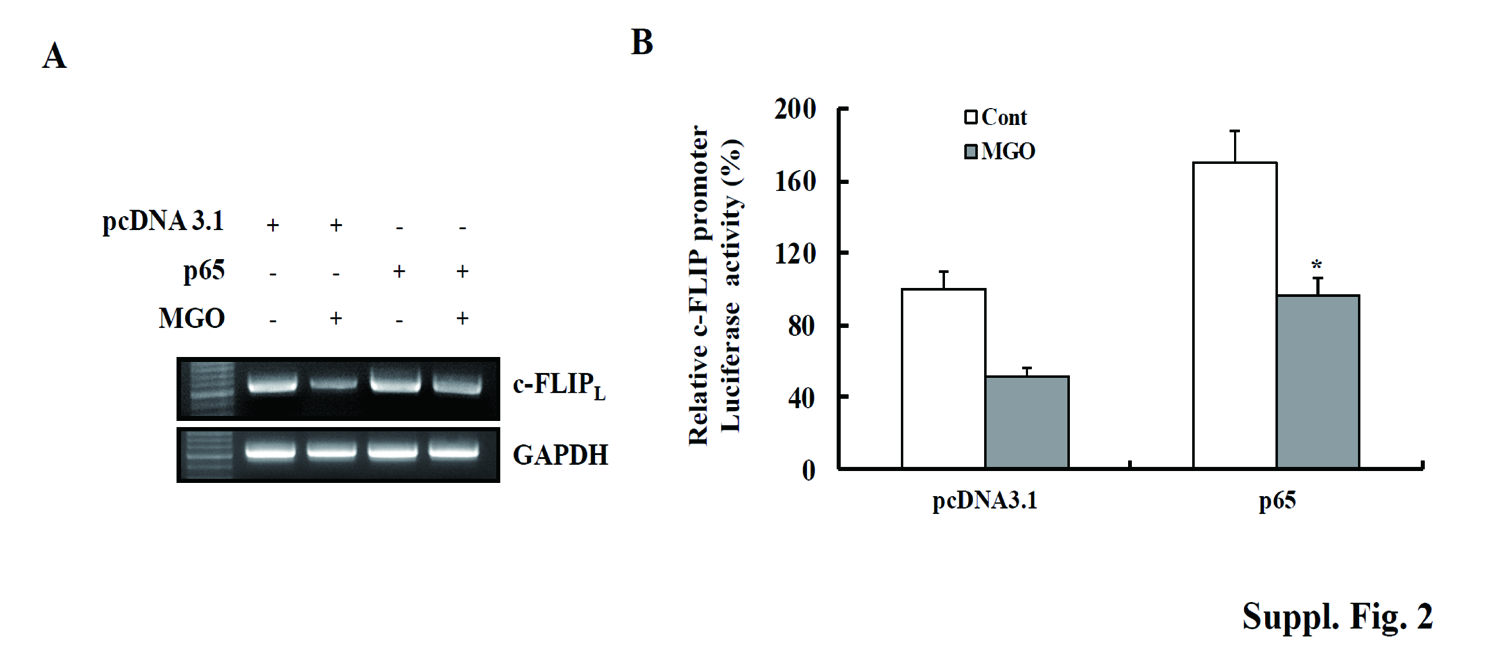

Supplement: Supplementary file 2 — Fig. S2 (A) RT‐PCR analysis of c‐FLIPL mRNA in EA.hy26 cells transfected as indicated. (B) EA.hy26 cells were transfected with a c‐FLIP promoter containing luciferase vector and then treated with MGO for 18 hrs. The cell lysates were assayed for the luciferase activity using a luminometer. The differences in transfection efficiency were normalized by cotransfecting with a LacZ‐containing plasmid. *P < 0.05 versus MGO‐treated pcDNA3.1 cells. [file JCMM-21-2720-s002.tif]

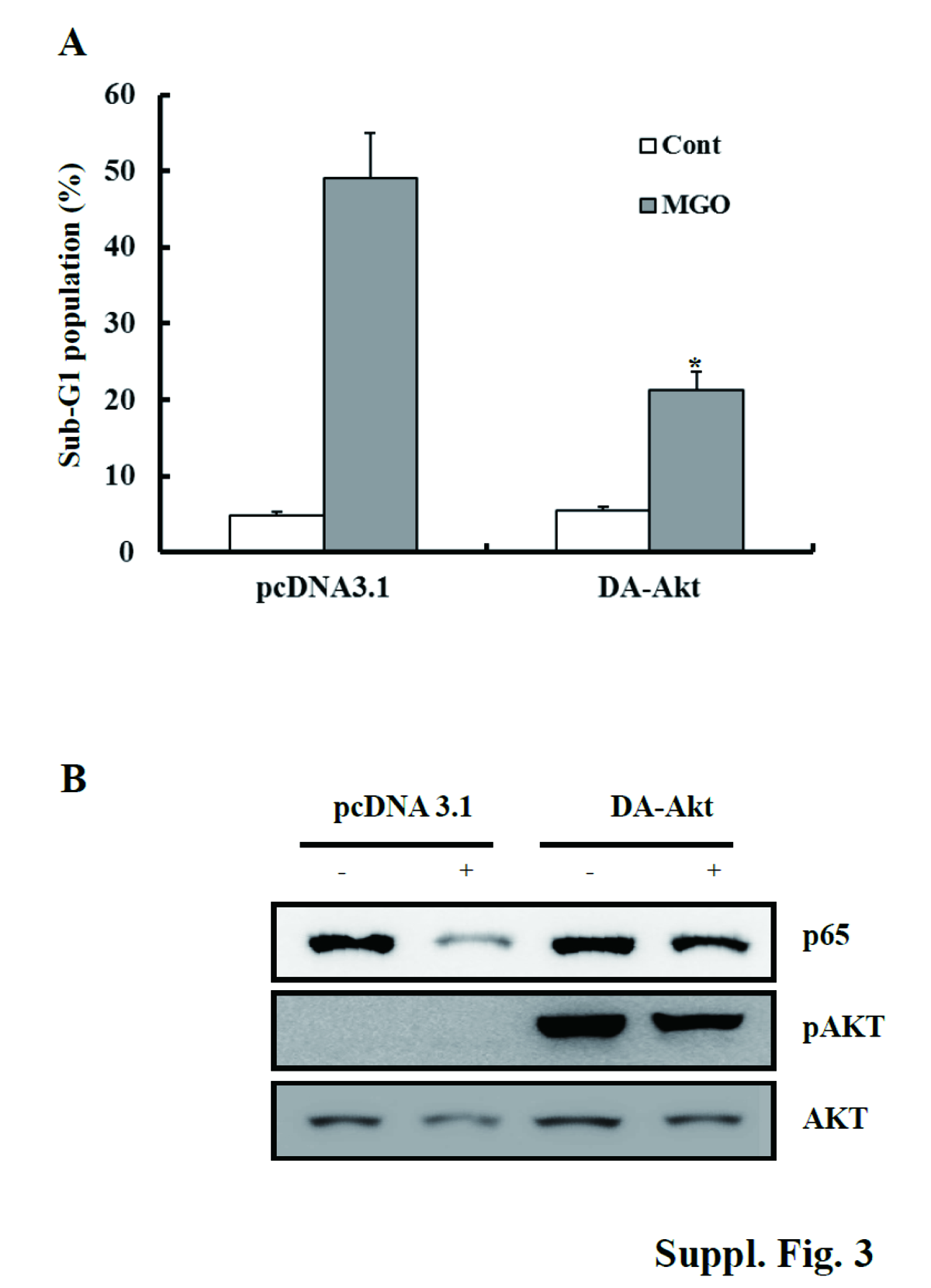

Supplement: Supplementary file 3 — Fig. S3 (A) EA.hy26/pcDNA3.1 and EA.hy26/DA‐Akt were treated for 18 hrs with MGO. Apoptosis was assessed by determining the proportion of cells in the sub‐G1 fraction by FACS. *P < 0.05 versus MGO‐treated pcDNA3.1 cells. (B) Equal amounts of cell lysates (40 μg) were electrophoresed and analyzed by Western blotting. [file JCMM-21-2720-s003.tif]
