# Supplementary material for: Methylglyoxal‐induced apoptosis is dependent on the suppression of c‐FLIPL expression via down‐regulation of p65 in endothelial cells
Source: J Cell Mol Med. 2017 Apr 26;21(11):2720–31. doi: 10.1111/jcmm.13188 (PMC5661116; doi:10.1111/jcmm.13188)
Supplement: Supplementary file 4 [file JCMM-21-2720-s004.docx]

**Supplementary Figure legends**

**Fig. 1.** (A) HUVECs were treated with the indicated concentrations of MGO. Equal amounts of cell lysates (40 μg) were subjected to electrophoresis and analyzed for caspase-8 and actin (for normalization) by western blotting. p55 indicates procapsapase-8. p43/41 indicate the cleaved capsapase-8 fragments. (B) HUVECs were transfected with si-c-FLIP_L_ with si-caspase-8 or si-Cont. Twenty-four hours after transfection, the cells were treated with MGO for 18 h. Apoptosis was analyzed as the sub-G1 fraction by FACS (left). *p < 0.05 compared to each MGO-treated si-Cont-transfected cells. Immunoblots for caspase-8 and actin antibodies (right). p55 indicates procapsapase-8. p43/41 indicate the cleaved capsapase-8 fragments.

**Fig. 2.** (A) RT-PCR analysis of c-FLIP_L_ mRNA in EA.hy26 cells transfected as indicated. (B) EA.hy26 cells were transfected with a c-FLIP promoter containing luciferase vector and then treated with MGO for 18 h. The cell lysates were assayed for the luciferase activity using a luminometer. The differences in transfection efficiency were normalized by cotransfecting with a LacZ-containing plasmid. *p<0.05 vs. MGO-treated pcDNA3.1 cells.

**Fig. 3.** (A) EA.hy26/pcDNA3.1 and EA.hy26/DA-Akt were treated for 18 h with MGO. Apoptosis was assessed by determining the proportion of cells in the sub-G1 fraction by FACS. *p<0.05 vs. MGO-treated pcDNA3.1 cells. (B) Equal amounts of cell lysates (40 μg) were electrophoresed and analyzed by Western blotting.
